# CT Volumetry of Convoluted Objects—A Simple Method Using Volume Averaging

**DOI:** 10.3390/tomography7020011

**Published:** 2021-04-13

**Authors:** Rani Al-Senan, Jeffrey H. Newhouse

**Affiliations:** 1 Department of Radiology, Pennsylvania State University, Hershey, PA 17033, USA; 2 Vagelos College of Physicians and Surgeons (VP&S), Columbia University, New York, NY 10032, USA; jhn2@cumc.columbia.edu

**Keywords:** CT, volumetry, segmentation, partial-volume averaging

## Abstract

Accurate measurement of object volumes using computed tomography is often important but can be challenging, especially for finely convoluted objects with severe marginal blurring from volume averaging. We aimed to test the accuracy of a simple method for volumetry by constructing, scanning and analyzing a phantom object with these characteristics which consisted of a cluster of small lucite beads embedded in petroleum jelly. Our method involves drawing simple regions of interest containing the entirety of the object and a portion of the surrounding material and using its density, along with the densities of pure lucite and petroleum jelly and the slice thickness to calculate the volume of the object in each slice. Comparison of our results with the object’s true volume showed the technique to be highly accurate, irrespective of slice thickness, image noise, reconstruction planes, spatial resolution and variations in regions of interest. We conclude that the method can be easily used for accurate volumetry in clinical and research scans without the need for specialized volumetry computer programs.

## 1. Introduction

Measuring the volume of a lesion or organ can be very useful for a variety of clinical, pathologic, and physiologic analyses. Since the introduction of computed tomography (CT) in the 1970s, there have been numerous methods proposed to estimate the volume of organs or tissues from CT images. The manual planimetric method of tracing the tissue’s boundary is perhaps the simplest approach but has its weaknesses [1,2,3,4]: it is tedious, prone to inter-user variation, has difficulty in identifying unsharp interfaces and is often inaccurate [5,6,7]. Other, more sophisticated approaches include:

- Utilization of a histogram of CT numbers to identify object size [8,9].
- A custom-made software has been used for this method [10].
- A semiautomatic method in which software interpolates an organ contour from a manually outlined contour in one slice [4].
- A curve fitting approach [11].
- Automatic segmentation of tissue of interest [3,12,13,14,15]. Automated segmentation methods include thresholding, region growing, morphologic techniques and watershed analysis [16].

Most computer-based volumetry programs ultimately depend on precise localization of the boundaries between the measured object and surrounding tissue. Threshold density values chosen to represent interfaces may vary [16] and produce differences in boundary location. Convolution of surfaces, especially when the interfaces are intricate on a small scale, produces severe partial volume effects and subsequent unsharp boundaries which cannot be precisely localized. As a result, there are often significant errors encountered with the use of these programs [16,17,18], and as assessed objects become smaller, such that the convoluted surfaces involve large fractions of their images, the problem worsens [18,19].

Other factors exacerbate these inaccuracies. Thick acquired slices produce more partial volume effects at interfaces, and are associated with greater errors [19]. Larger pixels and noise introduced by lower radiation doses produce uncertainty of boundary location, and thus also increase error.

If a structure has a uniform density, however, and is surrounded by a tissue which has a uniform but different density, it is possible to determine accurately the volume of the structure without identifying its surface, no matter how convoluted the interface between the structure and its surrounding tissue may be. The process, which we present in this paper, is relatively rapid and can be performed on any standard workstation without dedicated computer algorithms. Our aim was to test the accuracy of the method; to this end, we created and scanned a phantom with an intricately convoluted surface and compared its calculated volume with an independent volume assessment. We found the method to be highly accurate, even with considerable variations in slice thickness, field of view, radiation dose, region-of-interest (ROI) shapes, reconstruction planes and kernels.

## 2. Methods and Materials

### 2.1. Theory

The theory behind this method is based on the fact that the CT number in Hounsfield units (HU) for any region of interest (ROI) is equal to the weighted average of the HUs of the different materials contained in that ROI, which can be written as:

(1) $$\bar{\mathrm{HU}}={\mathrm{HU}}_{m1}\times f_{m1}+{\mathrm{HU}}_{m2}\times f_{m2}+{\mathrm{HU}}_{m3}\times f_{m3}+\ldots$$

where $\bar{\mathrm{HU}}$ is the average CT number of the ROI; subscripts *m*1, *m*2, *m*3,… refer to the different materials, and *f* is the fractional contribution of each material to $\bar{\mathrm{HU}}$. If more than two materials are involved, it may be impossible to determine the *f* for every material by only knowing the $\bar{\mathrm{HU}}$ and the HU for the different materials; histogram analysis or special software algorithms are required. However, if there are only two materials (or tissues); A and B, and material A is fully contained in (or mixed with) B which has a different HU, it is theoretically possible to determine the fraction of A and hence its volume using the values of both HU and area of an ROI (region of interest), which covers only these two materials. These two values are readily identified in any modern DICOM image viewer.

### 2.2. Phantom Construction and Scanning

A phantom was built to create an object whose shape would create CT images with highly convoluted indistinct borders and would have many voxels that would contain both the object and the surrounding substance. The substances were chosen to approximate the densities of human non-lipid-containing parenchymal organs and normal human fat. Around 750 spherical beads of different sizes, made of poly(methyl methacrylate) (PMMA), which has a density of 1.19 g/cm^3^, were used to create the central object. These spheres varied in diameters between 3.2 and 6.4 mm, with a total volume, measured by water displacement, of 35.5 cm^3^.

Pure petroleum jelly (Vaseline^®^, Unilever, Trumbull, CT, USA), which has a density ranging from 0.82 to 0.87 g/cm^3^ and a melting point between 40° and 50° Celsius [20] versus a 160° Celsius melting point for PMMA [21], was used to represent fat.

A portion of the petroleum jelly was kept at room temperature, at which it is semi-solid. The center of its superior surface was scooped from it, leaving a concave surface deeper in the middle than at the edges. The spherical beads were then placed on this concave surface, where they formed a conglomerate structure with multiple contact points among the bead surfaces. The remainder of the jelly was then melted and gently poured over and around the PMMA beads. The phantom was then kept at room temperature to allow the melted jelly to solidify. Gentle pouring of the melted jelly and slow cooling permitted solidification of the entire structure without trapping bubbles of air.

The phantom was scanned with a 64-slice LightSpeed VCT (GE, Milwaukee, WI, USA) CT scanner. Scan parameters were: 120 kV, 300 mA, 0.6 s rotation, pitch of 1.375. Axial images were reconstructed using a 14 cm reconstruction diameter, Standard kernel, ASiR (Adaptive Statistical Iterative Reconstruction) of 50%, and scan thicknesses of 5.0 and 0.625 mm. The scan was run three times.

### 2.3. Determining Volume of Spherical Beads

Images were viewed using Centricity PACS Radiology RA1000 Workstation and Exam Manager (GE Healthcare, Barrington, IL, USA).

**Figure 1:**
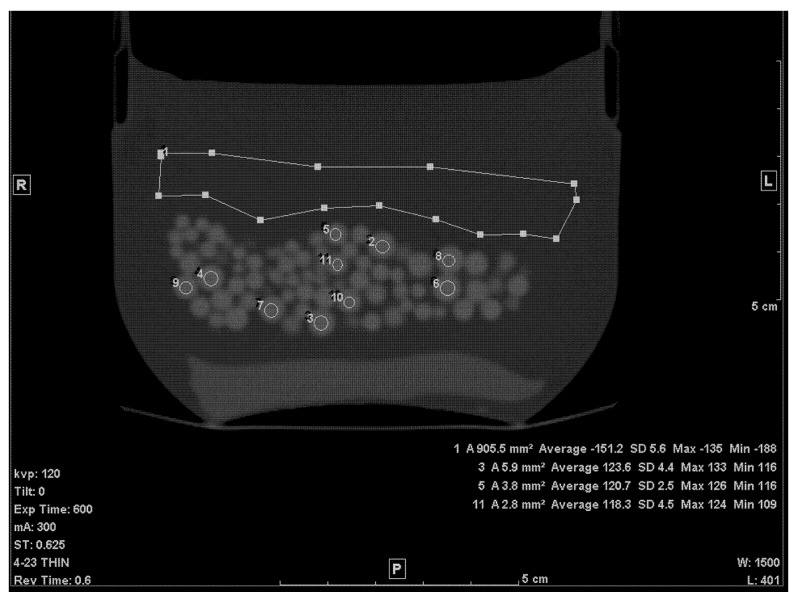
A thin slice (0.625 mm) of the scanned phantom with ROIs to determine average CT# of Lucite beads and Vaseline.

The first step of calculating the volume of the beads within the petroleum jelly was to establish the average CT densities of the petroleum jelly and beads. The thin (0.625 mm) slices were used for this purpose. The density of petroleum jelly was established by drawing a ≈ 10 cm^2^ region of interest (ROI) on a region containing only petroleum jelly (Figure 1). For PMMA beads, the average density was determined by drawing circular ROIs on ten beads, randomly selected, of various sizes (Figure 1). To avoid any volume averaging with petroleum jelly, thin slices (0.625 mm) were used and, in addition, the small ROI (≈1/4 to 1/3 the size of the bead) was drawn inside the bead in the slice where the bead appeared largest.

**Figure 2:**
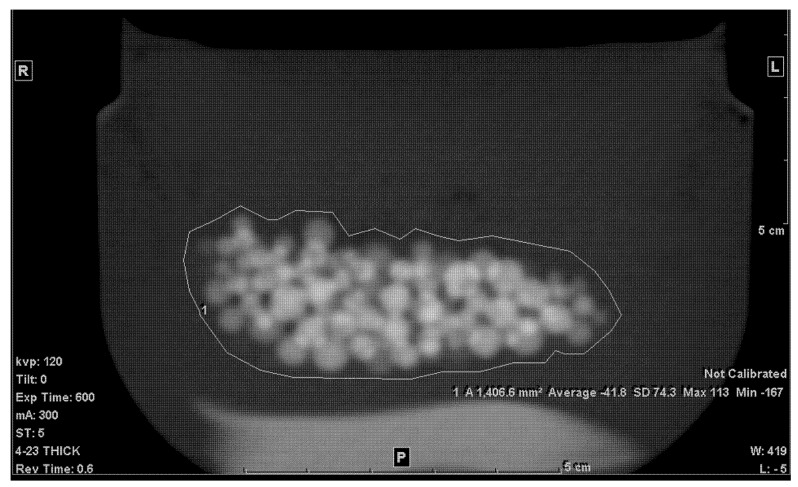
Freehand ROI of the mixture on a 5 mm-thick slice.

The second step was to draw an ROI in each of the slices that included any portion of the beads. A freehand ROI drawing option was used to ensure that no other pixels other than the jelly and the beads were included, as seen in Figure 2. For each slice, the mean density in Hounsfield units (HU), standard deviation (SD), and the area of the ROI in mm^2^ were shown on the workstation image. Both the mean density and area were recorded.

The final step was to estimate the volume (in cm^3^) of the beads. The principle used was that, for a given area of the mixture in any slice, the fraction (*f*) of the beads contained in this area may be estimated as:

(2) $$f=\frac{\bar{\mathrm{HU}}-{\mathrm{HU}}_{jel}}{{\mathrm{HU}}_{b}-{\mathrm{HU}}_{jel}}$$

where $\bar{\mathrm{HU}}$ is the average CT# of the ROI of the mixture, ${\mathrm{HU}}_{jel}$ is the average CT# of the petroleum jelly, and ${\mathrm{HU}}_{b}$ is the average CT# of the beads. This was calculated for all the slices containing the mixture.

Therefore, the volume in cm^3^ was calculated as:

(3) $$V=\frac{T}{1000}\times \overset{N}{\underset{i=1}{\sum }}A_{i}\times f_{i}$$

where *i* is the slice number, *T* is the slice thickness, *N* is the total number of slices which contained the mixture and *A* is the area in mm^2^. Since both the area and slice thickness were in mm, 1/1000 converts the result into cm^3^.

Relative error was used as the accuracy index of the calculated volume and was estimated as:

(4) $$Relative\;error\;\left(\%\right)=100\times \frac{\;V-actual\;volume}{actual\;volume}$$

### 2.4. Influence of Reconstructed Slice Thickness, Plane, FOV, Noise, Kernel, and ROI Size on Accuracy

To examine the influence of scanning and reconstruction parameters, the following tests were performed:

- Noise level: the scan was repeated with lowering the tube current to 10 mA.
- Slice thickness: in addition to the 5.0 mm thick slice, reconstruction was repeated using 0.625 and 2.5 mm slice thicknesses.
- Image plane: coronal and sagittal reformat planes were reconstructed from the 0.625-mm slice images.
- In-plane spatial resolution: the effect of this factor was evaluated in two ways; (i) the effect of pixel size was tested by changing the reconstruction FOV to 30 cm and 50 cm. (ii) reconstruction kernel was changed from standard to Lung (edge-enhancing) kernel.
- Dependence on ROI size: ROIs (on average) as large as double the area of the original ones were drawn
- Error in HU_bead_ and HU_jel_: The volume of the beads was calculated using values for the densities of the beads and/or background which were several digits above or below the values measured from the scans.

In each case, volume was then calculated using the same formulae (Equations (2) and (3)) given in the previous section.

## 3. Results

**Figure 3:**
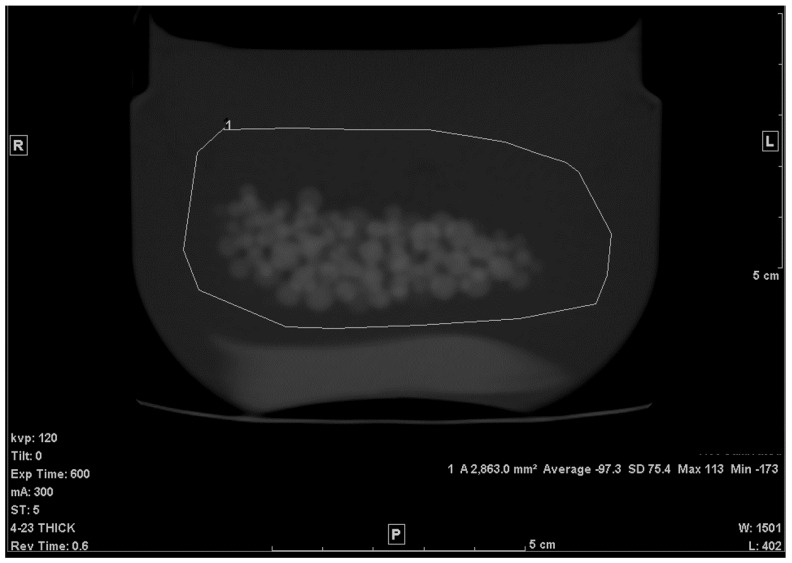
Large ROI (×2 the area of the original ROI).

The average CT density, measured from the 0.625 mm slice, for petroleum jelly at 120 kV was −149.7 +/− 5.3 HU. The PMMA beads’ average CT density with SD was 119.9 +/− 3.7 HU. The average volume from the three repeated scans with the 5.0 mm slice was found to be, on average, 34.6 cm^3^ and the error, therefore, was −2.5%. The same result was found with 0.625 mm slices, and a very slight difference was found with 2.5 mm slices; V = 34.7 cm^3^ (error: −2.3%). The very low-dose (high-noise) scan showed a slightly higher error (−3.7%), as shown in Table 1. The SD of a uniform area of petroleum jelly was our indicator of noise level (graininess) in the images. The SD in both original (low noise) vs. high-noise scans were, respectively, 4.6 HU and 22.3 HU. No noticeable change was found with either 30 or 50 cm FOV. A higher error was found with the Lung kernel images (−6.2%). It is worth mentioning that with this kernel, the average CT density of the beads and the jelly was, respectively, 137.6 (+/−7.5 HU) and −147 HU (+/−8 HU). The highest error was obtained with the large (double the size) ROI (Figure 3); the volume was 33.2 cm^3^ (−6.5%). Errors in both coronal and sagittal MPR were −3.7% and −3.6%, respectively. Table 1 lists the results.

**Figure 4:**
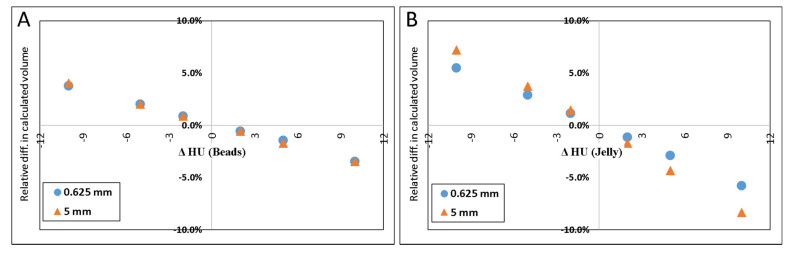

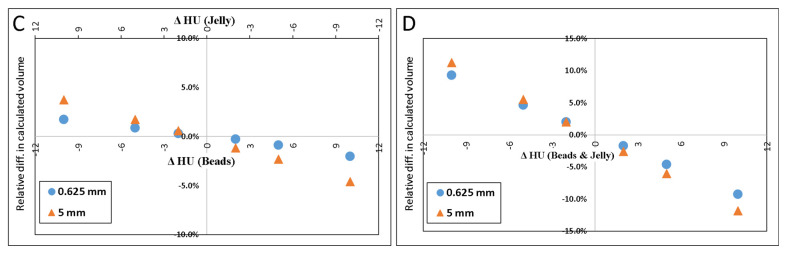
Scatter plot of the difference in calculated volume when HU of petroleum jelly or PMMA beads was changed. ΔHU indicates the difference in the HU. Scatter plot (**A**) shows the effect (in percent) of calculated volume when only HU*_b__ead_* was changed. (**B**) shows the result when only the HU*_j__el_* was changed. In (**C**), the plot demonstrates the result when either one was changed by one sign (+/−), and the other was changed by the opposite sign. When both materials’ HU were changed by the same sign the resulted effect on the calculated volume is shown in (**D**).

Figure 4A–D illustrate the relative errors when either or both of the materials’ HU differed from the measured values by +/−2, +/−5 or +/−10 HU from the values determined above (petroleum jelly: −149.7 HU, beads: 119.9 HU). The *y*-axis presents the % difference in the resulted volume relative to the volume given in Table 1 for both 0.625 and 5.0 mm slice thicknesses.

The results of simulated scenarios when the HU of the background material (jelly) and beads were changed by 2, 5, and 10 HU are demonstrated in Figure 4.

## 4. Discussion

In this paper, we present a simple approach to estimate the volume of a material randomly interspersed with, and fully contained in, another material. The method was originally proposed over forty years ago by Miller et al. [22], but we are unable to find any reference to its use since then. The results show high percent accuracy (within 3% for axial plane and <4% for coronal and sagittal planes). One major advantage of this method is its simplicity and the fact that a radiologist can easily apply it while looking at the images on a PACS workstation, that is, no segmentation software or histogram analysis is required. Furthermore, the results show no appreciable dependence on slice thickness; i.e., even if the object size was much smaller than the thickness of the slice, the volume result was accurate. The smallest bead size was 3.2 mm and the thickest reconstructed slice was 5.0 mm, yet the error was <3%. In the same manner, the results show no reliance on the pixel size of the reconstructed image.

The cause of even our small error is not known with certainty. It is possible that it was caused by air adherent to the surfaces of the beads; even though we could detect no bubbles, deposits of air too small to be visible in our scans could have reduced the average density of each slice, which would reduce the calculated volume of the beads. An error of this sort would be very unlikely to occur in clinical scans.

**Figure 5:**
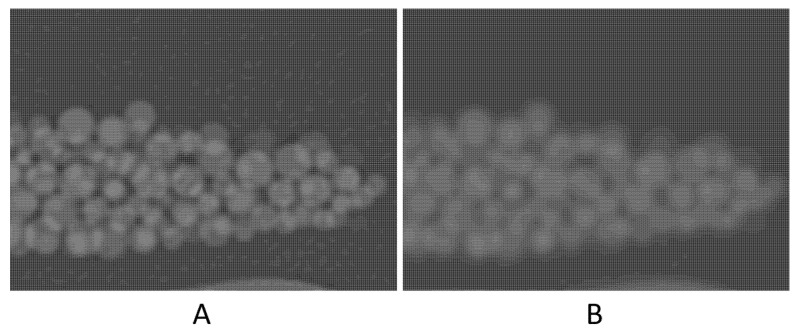
Zoomed-in images to compare the image appearance with Lung filter (**A**) and standard filter (**B**).

High noise level (four times higher than that of the original scan) seemed to have resulted in ~1% degradation in accuracy. The contrast-to-noise ratio (CNR) in the original scan is 50.8, as opposed to 12.1 in the high-noise scan. This 75% reduction in CNR, despite being large, had only a small (~1%) effect on the volume measurement accuracy. A noticeable decrease in accuracy was with the high-resolution (Lung) kernel; it changed to −6.2%. This kernel, like other edge-enhancing kernels, uses algorithms that alter CT numbers and noise texture in a non-uniform manner to preserve the high-resolution structures of the image^23^. Figure 5 shows the difference in the image appearance of a standard versus Lung kernels. The high-density variation in both the background (petroleum jelly) and beads is likely the cause of the relatively high error.

When the ROI was increased in area (Figure 3), the calculated volume appeared to decrease by a few percent. One reason for this change may be attributed to the fact that when the drawn ROI covered a larger area of the background material (petroleum jelly), the resulting $\bar{\mathrm{HU}}$ was further shifted toward the HU of the background and, hence, the fraction of petroleum jelly became larger (*f* became smaller). Therefore, one can conclude that the larger the ROI, the greater the change (or error) in the volume calculation.

The result of inaccurately determined HU, shown in Figure 4, which may also be inferred from Equations (2) and (3), indicates that inaccuracies in HU of the material of larger volume (in this case, it is the jelly) would result in a larger error in the calculated volume than inaccuracies in the HU of the material of a smaller volume.

Other possible sources of the errors include the variation in density in either material; the lower the homogeneity the lower the accuracy. This indicates that the error in applying this method also depends on Δ*ρ* (difference in density between the two materials); that is, for any given error in the average HU*_m_*_1_ and/or HU*_m_*_2_ the error of the calculated volume decreases as Δ*ρ* increases. In addition, helical reconstruction which is based on linear interpolation may contribute to a small degree of error, e.g., the relatively higher slice sensitivity profile in helical vs. step-and-shoot scan [23].

This method should be particularly useful in circumstances in which the organ or structure to be measured has a highly convoluted surface, especially when the convolutions are sufficiently small-scale that several may exist within the thickness of the scanned slice. In these situations, volume averaging effects may be sufficiently severe that any manual or computer-based edge-identifying method of volume determination is likely to be inaccurate. As an example, this phenomenon occurs when organs undergo irregular infiltration of their tissue with fat, which may occur in the muscle or pancreas.

When using the standard kernel, the results show an average error of 3%, as opposed to 7% in Miller’s study. The main possible reason for this difference is the improvements in scanners’ hardware and software since 1977. Other published methods have shown variations in accuracy. For instance, the percent error reported from using the planimetry approach was between 1% and 19% [1,2]. Manios et al. [10] reported an error ranging from 5% to 9% using the random marking method. Additionally, an error of 2% +/− 5.4% was found from the segmentation method [15]. Identification of the exact locations of the object–background interfaces may produce errors: with manual planimetry, the margins will appear to move as the window and level are changed in the display, and with segmentation algorithms they will move as the assigned density threshold locating them is changed. Our method avoids this type of error since the spatial location of tissue interfaces is not necessary.

A major limitation of this method is that it requires both the object to be measured and its surrounding tissue each be of uniform density. A common clinical circumstance requiring volume measurements occurs in oncologic imaging; most tumors are not of uniform density, either intrinsically or after contrast enhancement, and would not be accurately measured by this technique. Additionally, the tissue surrounding the object of interest may be heterogenous as well; for example, the tissue surrounding an intrahepatic tumor may include large vessels, dilated bile ducts and hepatic tissue rendered inhomogeneous by fatty infiltration and non-uniform contrast enhancement. Additionally, it is necessary to construct regions of interest which contain only two homogeneous tissues. This may be easy in certain circumstances; for example, to measure the volume of a homogenous spleen surrounded by homogeneous fat, it is not difficult to exclude large blood vessels in the splenic hilum. However, in other circumstances, such as that in which a single tumor invades more than one contiguous organ, fat and bone, it may be impractical. Nevertheless, there are situations in which two homogenous tissues can be isolated from other organs; for example, we have used it to quantify atrophy in the pancreas, and it should be useful to assess muscle atrophy when fat infiltrates muscle in random and intricate configurations. In patients with hepatic or renal polycystic diseases, relative volumes of cysts and surrounding organ parenchyma could be determined, as could volumes of endoleak cavities in patients with aortic endografts. This method could also be used to assess volumes of extracorporeal materials; for example, micro-CT of mineral deposition on small-scale scaffolding to create synthetic bone [24] could assess osteoblastic activity.

## 5. Conclusions

Our method of using partial volume calculations to find the volume of a material mixed with, and fully contained in, another material was experimentally validated as being highly accurate using a phantom of acrylic beads embedded in petroleum jelly. The method is robust; no large errors are introduced by changing slice thicknesses, regions of interest, reconstruction planes or pixel size; it can be easily employed without specialized software. The method should be useful in the volume assessment of structures with highly convoluted configurations when it and the surrounding tissues are of homogeneous and different densities. It is robust, simple, remains quite accurate even when slices are thick and reconstructed in planes other than the acquisition plane, and can be performed efficiently in the absence of specialized software.

## Figures and Tables

**Table 1:** Results of calculated volume with the standard parameters and with altered parameters.

Changed Parameter	Calculated Volume	Error
Standard (14 cm FOV, 5 mm slice, standard filter, axial images)	34.6	−2.5%
0.625 mm	34.6	−2.5%
2.5 mm	34.7	−2.3%
High noise (×4 times)	34.2	−3.7%
Lung Filter	33.3	−6.2%
Coronal	34.2	−3.7%
Sagittal	34.3	−3.6%
30 cm FOV	34.7	−2.3%
50 cm FOV	34.6	−2.5%
ROI of mixture is doubled in size	33.2	−6.5%
